# Bioaccumulation of metals in calanoid copepods by oral intake

**DOI:** 10.1038/s41598-019-45987-2

**Published:** 2019-07-01

**Authors:** Esther U. Kadiene, Baghdad Ouddane, Jiang-Shiou Hwang, Sami Souissi

**Affiliations:** 1 Université de Lille, CNRS, Univ. Littoral Côte d’Opale, UMR 8187, LOG, Laboratoire d’Océanologie et de Géosciences, 62930 Wimereux, France; 2 Institute of Marine Biology, National Taiwan Ocean University, 20224 Keelung, Taiwan; 3 Center of Excellence for the Oceans, National Taiwan Ocean University, Keelung, 20224 Taiwan; 4 Université de Lille, LASIR-UMR CNRS 8516, Equipe Physico-Chimie de l’Environnement, Bat. C8, 59655 Villeneuve d’Ascq, France

**Keywords:** Ecophysiology, Ecophysiology, Environmental impact

## Abstract

We demonstrated that oral intake of water by two calanoid copepods, *Pseudodiaptomus annandalei* and *Eurytemora affinis* takes place and has implications for their ecotoxicology. In the first experiment, copepods were exposed to a dyed medium, which allowed us to visually examine the possibility of water uptake by the copepod. We observed that both copepod species were taking in water orally and evacuated dye at different speeds. This exposure left concentrated dye in the guts of the copepods indicating adsorption into the gut epithelium. This was further demonstrated by exposing both copepod species independently to dissolved metals (Cd,17 µg/L; Cu,13.8 µg/L; Ni, 29.3 µg/L) and to dietary metals (Cd,18.8 µg/g; Cu, 35.3 µg/g; Ni, 32.5 µg/g). The results showed that although the concentration of dissolved metals they were exposed to were lower than those of the dietary metals, nevertheless, uptake of metals by both copepod species from the dissolved phase alone was substantially higher than from dietary exposure. This provides clear evidence to support our hypothesis that higher metal body burden observed in copepods exposed to dissolved metals than in those exposed to dietary metals is an implication of oral intake of water. *P*. *annandalei* showed higher excretion rate of metals when exposed to dissolved metals than *E*. *affinis*. However, the excretion rate of metals from both copepod species exposed to dietary metal was similar. We conclude here that both copepod species take in water orally. Our study further showed that metal uptake depends on the exposure routes and the uptake and excretion rates are dependent on the type of metals, amounts and the species.

## Introduction

Heavy metals are increasingly contaminating marine, brackish, and freshwater environments. Increased emission of heavy metals from anthropogenic activities increases their concentration in seawater, which enhances their bioaccumulation in the tissue of marine organisms and affects them through their toxicity^1^. The properties of toxic chemicals with respect to their water-solubility (hydrophobic or soluble) play a major role on how they interact with aquatic organisms. For example, when aquatic organisms are exposed to hydrophobic chemicals present in diet and water, diet-borne uptake becomes important since these chemicals are difficult to dissolve in water. However, even for a hydrophobic chemical like 4-nonylphenol, it was suggested that major uptake may also occur through water in the amphipod study^2^. This makes water an important uptake source for toxicants. Processes involved in the water-borne uptake of toxic chemicals include filtration, passive or facilitated diffusion, active transport or phago/pinocytosis^3^.

Copepods accumulate metals by assimilating them from their food or by absorbing them from water. Furthermore, the uptake pathway can determine its internal distribution and toxic action^4^. Several studies proposed that direct uptake of metals from water occurs by either adsorption to cell, tissue, organ, or organism surfaces, or via the absorption across cell membranes or organ epithelia such as the gill and/or gut^5–7^. Other studies showed that the accumulation of metals such as cadmium from water is higher than from food^4,8–11^. Cailleaud *et al*.^12^, suggested that pollutant uptake by planktonic species is governed by particular mechanisms and not only by adsorption and equilibrium partitioning between water and organisms. Gomes *et al*.^13^, indicated that uptake of toxic chemicals such as Estrone in *Daphnia magna* via the trophic route is likely to be less significant compared to bioconcentration from the aqueous medium.

Copepods are essential trophic links in marine food webs. Therefore, they can be a major source for biomagnification of toxic pollutants in aquatic food webs^14,15^. Feeding behavior has long been studied in calanoid copepods and their mode of feeding can be passive or active. They can switch between the two modes at intervals depending on the composition of their food^16,17^. Copepod feeding involves generating feeding currents by the beating of locomotory appendages, and capturing of food items that arrive with this current (suspension feeding)^16,18,19^, or ambush feeding, where passing preys are detected and captured in surprise attacks^20,21^, or when food particles collide with feeding appendages^22^. During feeding, the first three mouth appendages (antennae, mandibular palps and maxillules) create a backward motion of water with a metachronal beating pattern, and an asymmetrical vortex system is created on the ventral side of the animal^23^. The motion and feeding behaviour shown in the foraging tactic of *Clausocalanus furcatus* explores small volumes of water rapidly^24^. Koehl and Strickler^25^, showed that calanoid copepods do not strain algae out of the water as previously reported^26–29^. Rather, they flap four pairs of feeding appendages to propel water past itself and use its second maxillae to capture selectively parcels of that water containing food particles, which are then, pushed into the mouth by the endites of the first maxillae.

The study of copepod behaviour and feeding strategies has been of ecological importance for understanding the role of zooplankton in carbon and energy transfers through the aquatic food web and how these behaviours enable them to utilize different ecological niches. Since feeding pattern of copepods could involve water intake^25^, the present study demonstrated that oral intake of water by two calanoid copepods, *Pseudodiaptomus annandalei* and *Eurytemora affinis* takes place and has implications for their ecotoxicology. Our hypothesis is that metal uptake from water is a more important route in the bioaccumulation of metals than through dietary route because of oral intake.

## Results and Discussion

Oral intake was tested in both males and females of *P*. *annandalei* and *E*. *affinis* copepods. However, only the videos of females of each copepod species were presented. Moreover, the observed results of oral intake of water were the same in both sexes of the two copepod species.

**Figure 1:**
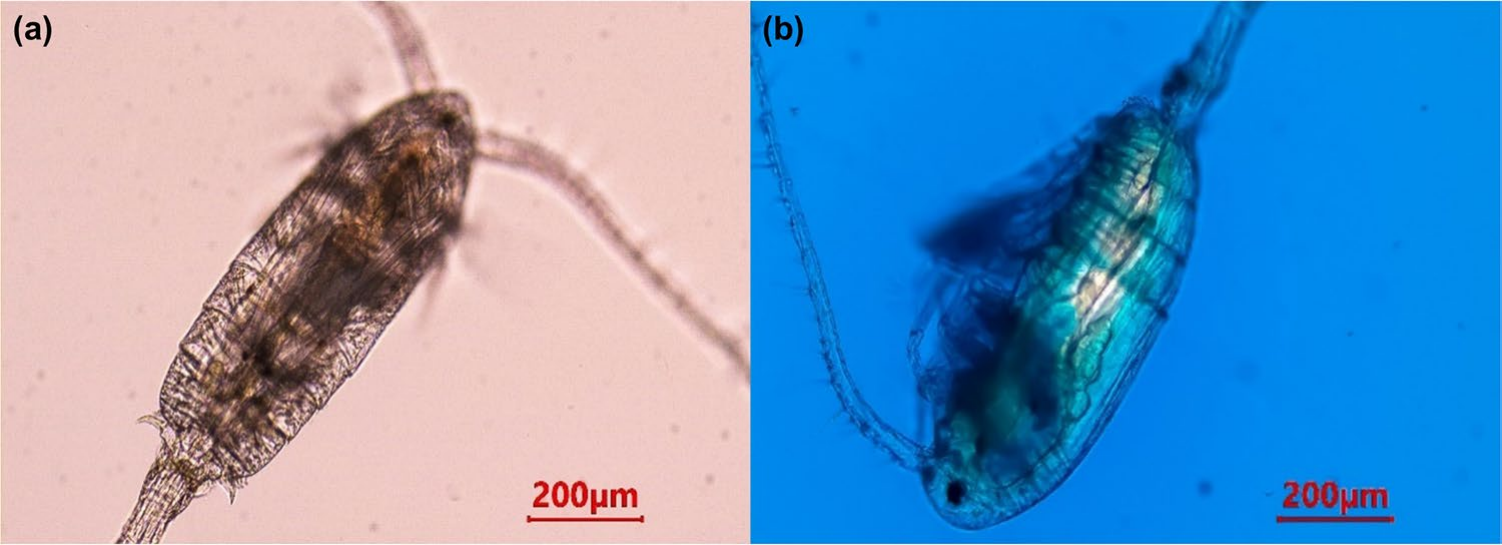
Non-ovigerous female of *Pseudodiaptomus annandalei* before exposure (**a**), showing a clear gut and during exposure to dye (**b**).

**Figure 2:**
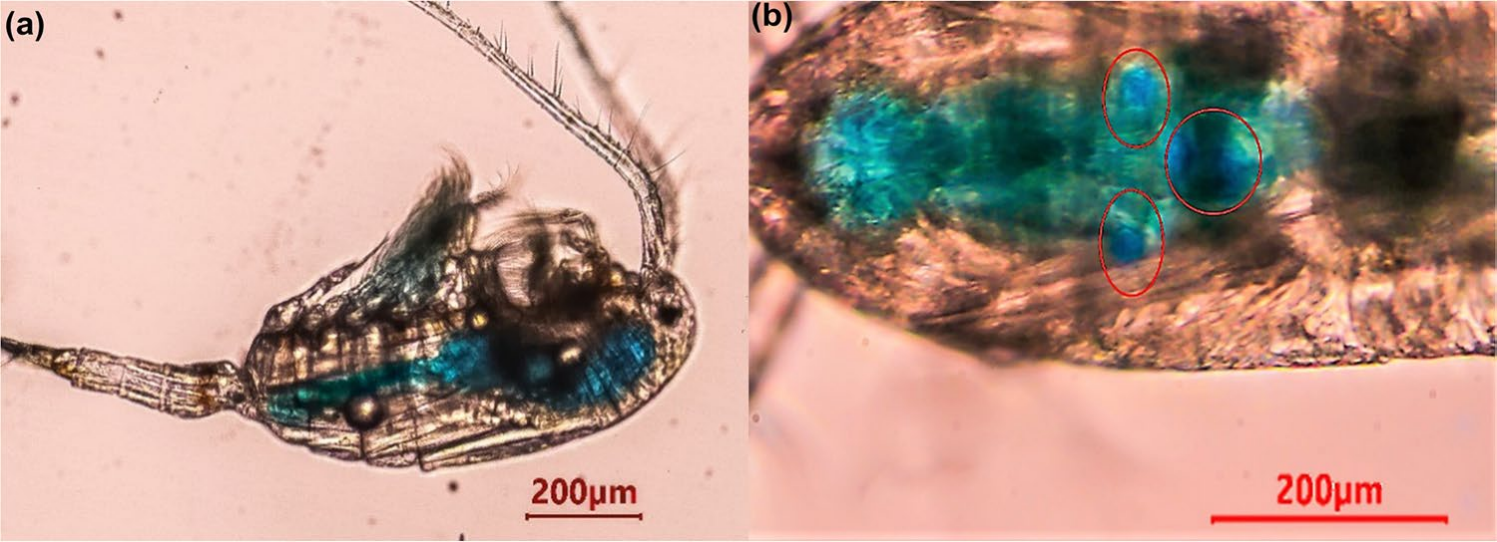
Non-ovigerous female of *P*. *annandalei* after exposure to dye, showing dye color in the gut (**a**), and patches of the dye concentrated around the midgut (**b**).

**Figure 3:**
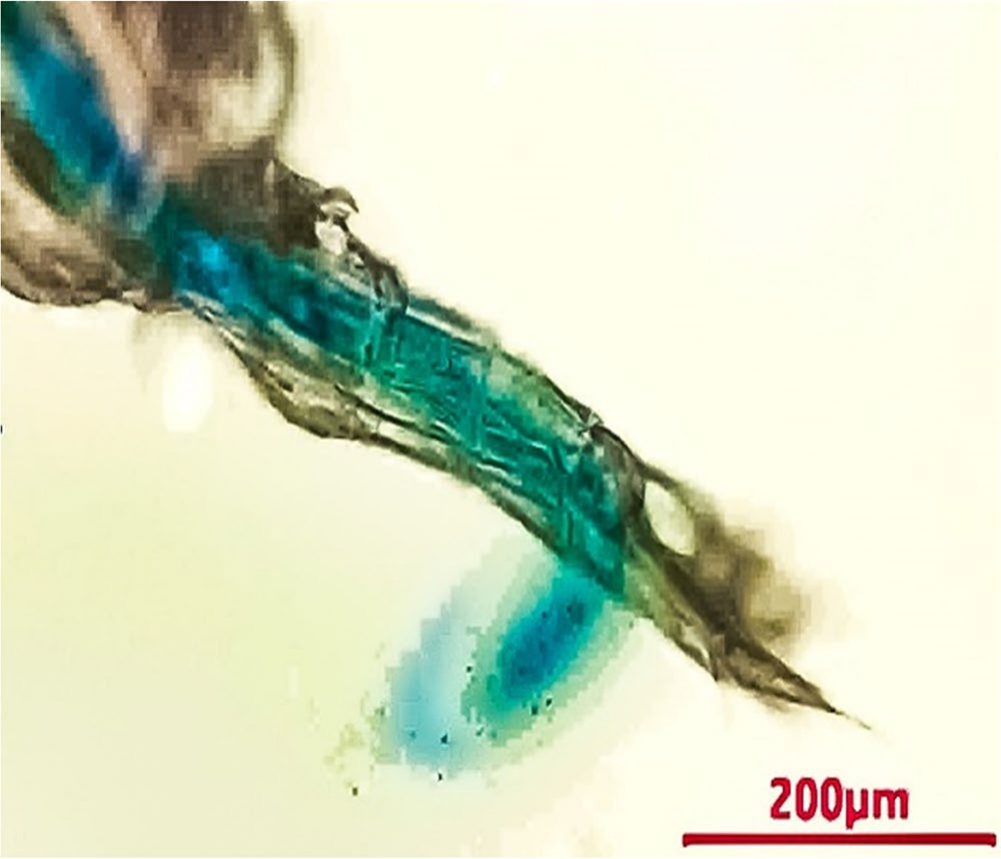
Non-ovigerous female of *P*. *annandalei* after exposure to dye, showing movement of the dye towards the urosome and excretion from the anus after ejection of a fecal pellet.

*P*. *annandalei* copepod kept unfed for more than 24 hours showed a clear gut (Fig. 1a) and after few minutes of adding dye to the medium, we observed that the dye was taken up and kept in the midgut. With increased water intake, the gut dilated (Fig. 1b). *P*. *annandalei* was observed to move the dye toward the hindgut for excretion (see Supplementary Movie 1). Figure 2 shows the dye contained in the copepod gut after the dye medium was replaced by clear water, although some amount of dye solution was excreted in the process. Approximately 30 minutes after the transfer of copepods to clear medium, further movement of the dye towards the hindgut was observed. The movements were aided by peristaltic contractions and forward and backward movements. Figure 3 shows large amounts of dye solution being excreted from the anus of the copepod following the egestion of a faecal pellet. It appeared that more ambient water was taken in orally (indicated by the increased size of the gut and lighter colour of the dye in the gut) (see Supplementary Movies 1 and 2).

Several studies showed that copepods create feeding currents for trapping and selectively taking in food particles into their mouth opening^24,30,31^. Fox^32^, thought that a continuous rhythmic swallowing of water through the mouth was part of the feeding mechanism of filter-feeding or vortex-feeding Crustaceans. Since the limbs are continuously collecting unicellular algae or detritus suspended in the water, it must be swallowed continuously. Food particles were not present in the medium in the first experiment and even though they did not often employ the use of their appendages, the dye solution was still taken in with each movement of the gullet. To observe them under the microscope without the copepod moving too fast away from the field of view, the surrounding water was reduced after few minutes to a sufficient volume; hence, less movement of the appendages were observed. Therefore, the peristaltic contractions of the gut and the gulping movement of the labrum during the vibratory movement of their appendages and even without their movement, brought water into the copepod as indicated by the dye. This implies oral intake of water by the copepod may not only be a feeding mechanism. Fox^32^, observed that in most crustaceans that he studied, the uptake of water was continuous, rapid and vigorous and the gulps of water were large. In the first experiment, the coloured water could easily be seen in the gut because the copepod is transparent enough to show the differences before, during and after staining (Figs 1 and 2). Fox^32^, also observed that the rhythmic oral intake of water by the cladocerans *Daphnia* and *Limnadina* showed each gulp been passed down the gullet corresponding to a movement of the jaws and muscle contractions. Weismann^33^, described the function of oral drinking as a respiration process. Moreover, Fox^32^, described it as a mechanism of feeding, stretching the muscles of the gut wall. The contractions maintained by the hydrostatic pressure of water pumped into midgut mixes food and digestive enzymes. Defecation occurs when this pressure rises to a certain level, forcing the food in the midgut back towards the rectum. In the present study, similar rhythmic oral water uptake was observed (see Supplementary Movies 1 and 2).

**Figure 4:**
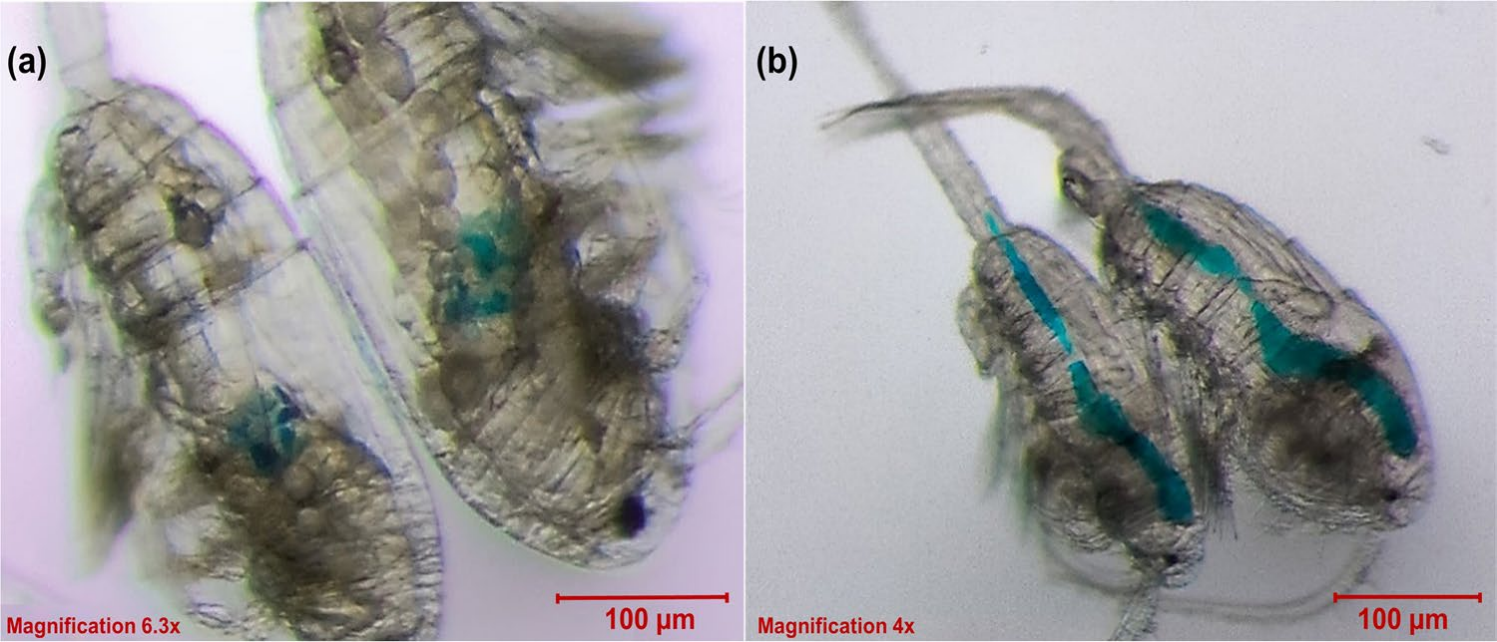
*Pseudodiaptomus annandalei* (**a**) and *Eurytemora affinis* (**b**) male and female copepods showing the dye stain (blue) concentrated in their midgut after 1 hr in clear water, following dye exposure.

A dye test by Fox^32^, demonstrated that *Daphnia* after exposure to a dilute solution of bromo-thymol blue or nigrosin for a few hours, the dye was concentrated about 250-fold in the intestine and the process of accumulation was rapid. Similarly, we observed dye-accumulation in our test (Fig. 2b (red circles)). The explanation for this phenomenon was that water was withdrawn through the gut wall from the solution. However, the dye concentrating around the midgut indicates adsorption by the gut epithelium. In experiment 1, another test was done to check the rate of dye evacuation in *P*. *annandalei* and was compared with *E*. *affinis*. Following their exposure to the dye solution, they were placed in clear medium and left for approximately 1 hour. When observed under the microscope, the dye solution in the gut of *P*. *annandalei* was cleared out except for the dye colour concentrated around the midgut (Fig. 4a, see Supplementary Movie 1). Whereas in *E*. *affinis* large remnants of dye solution could still be observed (Fig. 4b, see Supplementary Movie 2). *P*. *annandalei* is a tropical species that is cultured in the laboratory at temperatures ranging from 25° to 28°, whereas *E*. *affinis* is a temperate species cultured in the laboratory at temperatures ranging from 18° to 20°. It has been reported that temperature is positively correlated with gut evacuation rate^34,35^, this could explain why *P*. *annandalei* showed a faster rate of dye evacuation from the gut than did *E*. *affinis*. Gut contamination is a major source of variation in measured whole-body concentrations of several elements^36^. It has been demonstrated that assimilation efficiency of trace elements increased with longer gut passage time^10,37–39^. Moreover, the efflux rate of metals was higher following uptake from food than uptake from the dissolved phase^4^. In an egestion study with *Daphnia magna* and *Chironomus riparius*, Scherer *et al*.^40^, found that an exposure to food led to a shorter gut evacuation period of polystyrene spheres. This could imply that with a shorter gut evacuation time, the possibility of reducing or removing toxic pollutants is high. Therefore, the longer the organisms are exposed to contaminants especially from the dissolved phase, the more the contaminants are accumulated^41^, consequently increasing their toxicity^42–44^.

**Figure 5:**
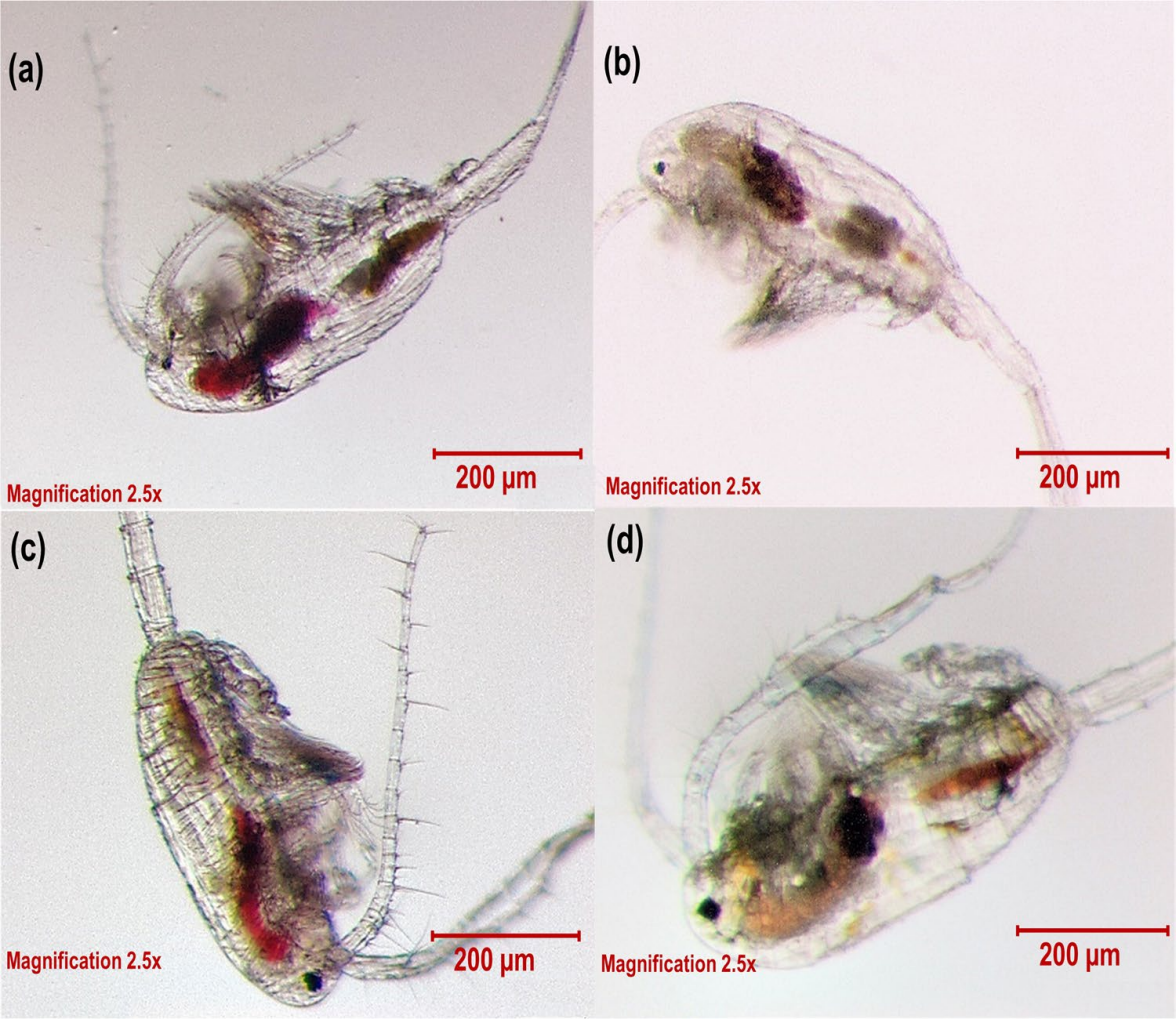
*Eurytemora affinis* female copepod few minutes after feeding with *R*. *salina* (**a**) and few hours later after last feeding. (**b**) *Pseudodiaptomus annandalei* male copepod few minutes after feeding with *R*. *salina* (**c**) and few hours later after last feeding(d).

Figure 5a,c shows *E*. *affinis* female and *P*. *annandalei* male copepods with algae *Rhodomonas salina* in their guts in the second experiment few minutes after feeding. If copepods were to selectively take in food particles into their mouth as previously reported^24,30,31^, then it may take a longer time for this amount of algae, indicated by red colour, to fill their guts. However, because the density of the algal cells was high in the medium, the resulting coloration (volume) of algae in their gut within few minutes of feeding (Fig. 5a,c) together with the dye intake in the first experiment without the presence of algae implies that water is taken into the copepods orally. In addition, after few hours of feeding *R*. *salina* to the copepods, discoloured food particles were observed in the gut (foregut). However, there were concentrations of red pigments possible digested or absorbed from the fed algae located in the midgut (Fig. 5b,d), similar with the dye concentrated in the copepods from the first experiment (Figs 2b (red circles) and 4a). Similar absorption might also take place in the case of metals^5–7^.

**Figure 6:**
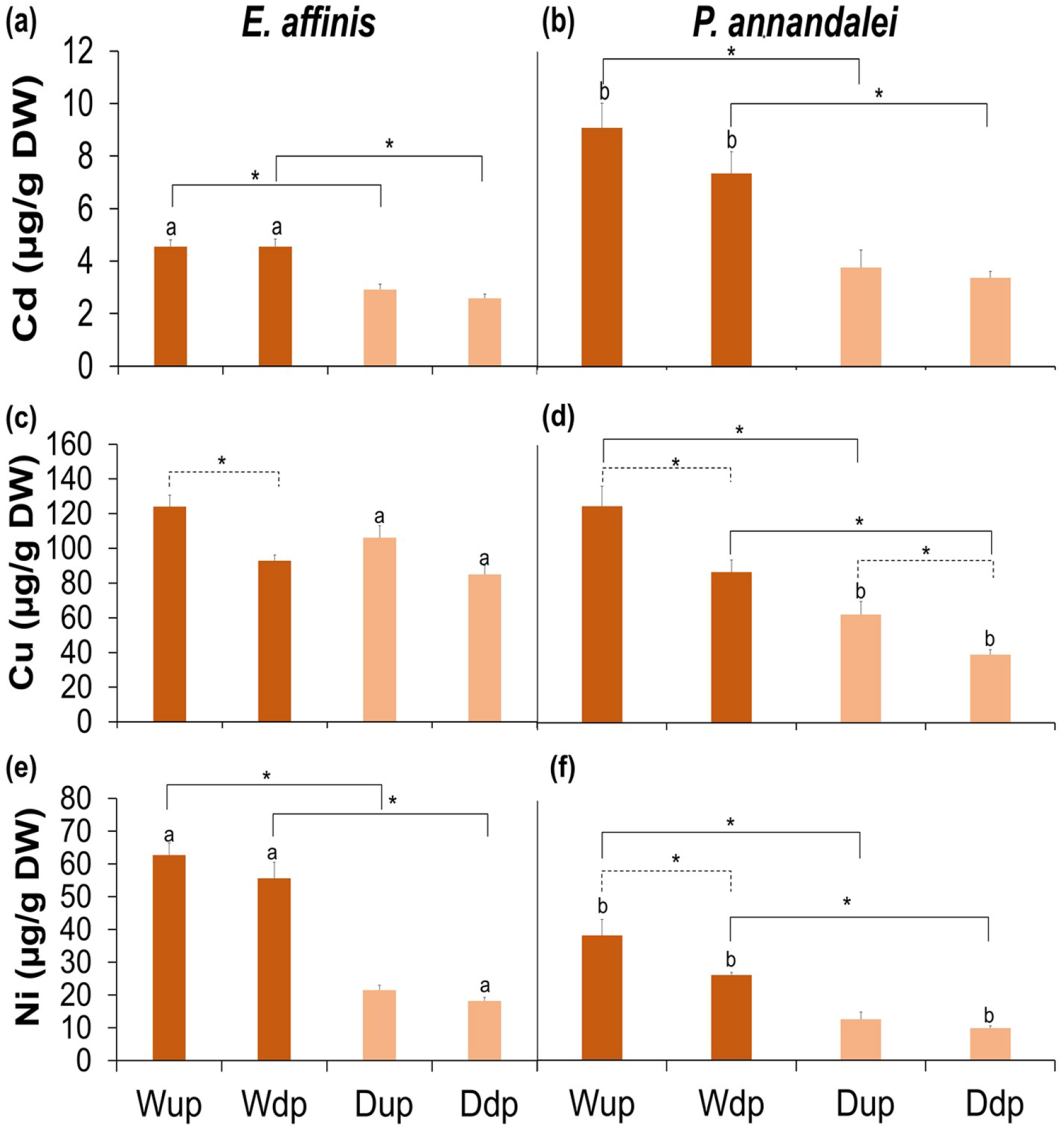
Concentration of metals in copepod after 4 hrs. uptake (Up) and after 2 hrs. depuration (Dp) from water (W) and diet (D: contaminated algae). Significant differences at *p* < 0.05 after uptake from water vs uptake from diet and after depuration from water vs depuration from diet exposures are presented as smooth brackets and asterisks (*) in *E*. *affinis* and *P*. *annandalei*. Significant differences at *p* < 0.05 after uptake from water vs depuration from water and after uptake from diet and vs depuration from diet exposures in *E*. *affinis* and *P*. *annandalei* are represented as broken brackets and asterisks (*). Significant differences at *p* < 0.05 after uptake from water and diet and after depuration from water and diet between *E*. *affinis* and *P*. *annandalei* are represented as alphabet a and b.

The toxicity of environmental pollutants to aquatic organisms depends among others, on route of exposure or entry^45^. It is commonly assumed that heavy metals enter copepods passively through diffusion across biological membranes^46–49^. Our first experiment demonstrated that dye was taken in by the copepods orally in large amounts. Therefore, the implication of oral intake of water by copepods in this study shows that dissolved metals besides taken in through membranes, could also be actively taken in larger amounts orally. We demonstrated this in the second experiment, by exposing the copepods to a mixture of metals in water only and through their diet with similar concentrations. We observed that metal uptake from the dissolved phase was significantly higher (*p* < 0.05) than metal uptake from the contaminated diet in both copepod species, even when the exposed concentrations in water was lower than those bioaccumulated in the diet (Fig. 6). Similar findings have also been reported^4,8–11,50^. This could be a result of oral intake of the medium, since this intake is frequent and in large gulps, they are constantly being exposed to the metals in the water.

In the first experiment, it took longer for *E*. *affinis* to clear out the dye solution and in the second experiment, during depuration, metals excreted from *P*. *annandalei* copepods were more than those excreted from *E*. *affinis* copepods exposed to dissolved metals. However, metals excreted from both copepod species exposed to dietary metal were similar (Table 1). Since oral intake of water is continuous, after been transferred to a clean medium, metal concentration decreased because clean water is exchanged with the contaminated water. Nevertheless, the amount of metals retained after 2 hours of depuration from exposure to water were still significantly higher than the metals retained from dietary exposure (Fig. 6). Uptake and excretion of metals by copepods can be specific to the kind of metals they are exposed to^51–53^. In this study, the order of the concentration of the metal mixture in dissolved phase that the copepods were initially exposed were Ni > Cd > Cu, however, in both copepod species, the order of highest metal uptake were Cu > Ni > Cd. And the order in which the mixed metals were bioaccumulated in the algae fed to the copepods were Cu > Ni > Cd, and the same order was taken up by both copepod species in terms of concentrations. Although, the concentration of copper (Cu) in the dissolved metal mixture at the beginning was the lowest (13 µg/L) among the 3 metals, however, it was the metal with the highest uptake from the dissolved phase. Moreover, the same concentration order of metals in the diets was in both copepod species. In addition, Cu was considerably the most excreted in both copepod species exposed to metals in water, and Cd the least. The lower gut evacuation rate of *E*. *affinis* could possibly make them more sensitive to metal toxicity. Kadiene *et al*.^44^, reported that *P*. *annandalei* was more tolerant to metal toxicity than *E*. *affinis* exposed to cadmium. This could further support the idea that physiological characteristics of copepods could affect the tolerance levels of pollutant toxicity.

**Table 1:** Difference between the concentration of metals taken up by *Eurytemora affinis* and *Pseudodiaptomus annandalei* copepods (µg/g DW) after 4 hours’ exposure to mixture of dissolved metals (water) (Cadmium (Cd); Copper, Cu; Nickel (Ni)) and dietary metals (Diet) and the concentration left in both copepods (µg/g DW) after 2 hours’ depuration in uncontaminated water.

	Water		Diet	
	*E*. *affinis*	*P*. *annandalei*	*E*. *affinis*	*P*. *annandalei*
Cd	0.03 ± 0.00	1.74 ± 0.01	0.34 ± 0.02	0.40 ± 0.01
Cu	31.20 ± 3.37	37.92 ± 4.80	21.29 ± 1.18	23.27 ± 4.57
Ni	7.08 ± 0.06	12.25 ± 4.23	3.37 ± 0.30	2.75 ± 0.50

Values are mean ± s.d.

In conclusion, we state here that both copepod species, *P*. *annandalei* and *E*. *affinis*, take in water orally. We suggest that this biological characteristic has an implication for the active accumulation of dissolved metal. In addition, the higher rate of gut evacuation shown by *P*. *annandalei* could be an adaptive mechanism of excretion of toxic pollutants. Our study showed that metal uptake depends on the exposure routes and the uptake and excretion rates are dependent on the type of metals, amounts and the species.

## Materials and Methods

In order to demonstrate the oral intake of water by copepods, we first added a local food dye solution (blue colour) dissolved (see Supplementary Movies 1 and 2) in water with male and non ovigerous female of *Pseudodiaptomus annandalei* and *Eurytemora affinis* copepods. They were unfed for more than 24 hours before the start of the experiment. The blue dye was composed of distilled water, propylene glycol, FD&C Blue 1, FD&C Red 40, and 0.1% propylparaben (preservative). This allowed us to visually examine the possibility of water taken up by the copepod in an attempt to better understand the process of metal bioaccumulation in copepods. After introducing the dye, they were left for five minutes to allow dyed water to be taken up. The copepods were then rinsed to remove the food dye and placed in another clean medium to examine the intake and ejection rate of the dye. The copepods were observed live under the microscope (Olympus BX51 and SZX10, Tokyo, Japan) and the behaviour was recorded by video.

In the second experiment, we investigated the implication of oral intake of water by copepods in metal bioaccumulation. Both *E*. *affinis* and *P*. *annandalei* copepods were filtered through 200 µm mesh filter (contained large copepodids and adults) from a large stock culture, and acclimated to the experimental conditions. *E*. *affinis* were cultured at 19 °C ± 1 and *P*. *annandalei* at 26 °C ± 1 and in a medium of salinity 15 for both copepod species. The copepods were concentrated in a beaker at a fixed volume. After homogenizing, equal amounts were distributed randomly into 5-liter beakers containing 4 litres of the treatment medium in duplicates.

Both copepod species were exposed to a mixture of 3 metals, copper (Cu), nickel (Ni) and cadmium (Cd) in the water and through their diet. *E*. *affinis* and *P*. *annandalei* were both exposed to the same sublethal concentrations, approximately 1/5^th^ 96 hr LC50 of each metal^54^ in the water (analysed concentration: Cd, 17 µg/L; Cu, 13.8 µg/L; Ni, 29.3 µg/L). The algae used was *Rhodomonas salina*, cultured with the mixture of 10x the 96 hr LC50^54^ of each of the above metals in Conway media (without EDTA) for 4 days before they were fed to the copepods, in other to achieve similar amount of metal as in the water exposure. The bioaccumulated concentration of the metals analysed in the algae were Cd: 18.8 µg/g; Cu: 35.3 µg/g; Ni: 32.5 µg/g (Table 2). Both copepod species were exposed to the mixture of metals in water only (and fed uncontaminated algae) and also to a mixture of metal contaminated algae only. They were sufficiently fed in both conditions twice at 1 hour intervals. The algae were centrifuged and rinsed with distilled water to remove all the metals in the culture water, to allow for metal uptake only from the contaminated diets. Approximately 4 hours later, half of the culture volumes were collected and filtered through 200 µm mesh filter to remove unwanted particles and then filtered through high quality filter papers to retain the copepods. The other halves were filtered through 200 µm mesh filter and transferred to new media without food and without contamination for approximately 2 hours. After which, the copepods were collected as before. Filter papers containing copepods and algae were dried in the oven at 70 °C for 72 hours and water samples were preserved with pure nitric acid for further analysis. Copepod samples were mineralized with 3 ml of ultrapure nitric acid (HNO_3_) at 105 °C for 2 hours in a hotplate. After dilution with pure water, inductively coupled plasma optical emission spectrometry (ICP-OES) was used to analyse the metal concentrations in the copepod, algae and water samples. Data were expressed as the mean ± standard deviation (SD). Multiple comparisons between means were made by one-way ANOVA for identification of the statistically distinct groups within each copepod species. Then, Student t-test was applied to compare the uptake of metals and the residual metals after depuration between both copepod species. Significant differences were accepted for *p* < 0.05. The statistical analyses were performed using SPSS, v.18.0 (SPSS Inc., Chicago, IL, USA).

**Table 2:** Metal concentrations (µg/L) in water before exposure, after 4 hours’ exposure to mixture of dissolved metals (Water) (Cadmium (Cd); Copper, Cu; Nickel (Ni)) and dietary metals (Diet) and after 2 hours’ depuration in uncontaminated water.

Water (µg/L)				Diet (µg/L)		
	Initial conc. in water	After 4hrs. Uptake	After 2hrs. Depuration	Initial conc. in algae (µg/g)	After 4hrs. Uptake	After 2hrs. Depuration
***E***. ***affinis***						
Cd	16.90 ± 0.14	16.50 ± 0.21	0.20 ± 0.07	18.82 ± 2.02	0.20 ± 0.14	0.20 ± 0.00
Cu	12.70 ± 1.48	10.90 ± 0.64	1.50 ± 0.28	35.27 ± 7.22	1.60 ± 0.00	1.70 ± 0.14
Ni	29.10 ± 0.31	26.83 ± 0.85	<DL	32.45 ± 1.09	0.85 ± 0.35	0.50 ± 0.14
***P***. ***annandalei***						
Cd	17.10 ± 0.07	16.80 ± 0.11	0.30 ± 0.04	18.82 ± 2.02	0.25 ± 0.07	0.15 ± 0.07
Cu	14.80 ± 0.74	10.00 ± 0.32	1.10 ± 0.14	35.27 ± 7.22	1.25 ± 0.35	1.80 ± 0.42
Ni	29.53 ± 0.15	28.03 ± 0.42	0.90 ± 0.30	32.45 ± 1.09	1.00 ± 0.14	0.40 ± 0.42

## Supplementary information

Oral intake of water by the copepod Pseudodiaptomus annandalei

Oral intake of water by the copepod Eurytemora affinis
